# Comparative metabolomics reveals the metabolic variations between two endangered *Taxus* species (*T. fuana* and *T. yunnanensis*) in the Himalayas

**DOI:** 10.1186/s12870-018-1412-4

**Published:** 2018-09-17

**Authors:** Chunna Yu, Xiujun Luo, Xiaori Zhan, Juan Hao, Lei Zhang, Yao-Bin L Song, Chenjia Shen, Ming Dong

**Affiliations:** 1 College of Life and Environmental Sciences, Hangzhou Normal University, Hangzhou, 310036 China; 2 Zhejiang Provincial Key Laboratory for Genetic Improvement and Quality Control of Medicinal Plants, Hangzhou Normal University, Hangzhou, 310036 China; 3 Department of Plant Pathology, Washington State University, Pullman, WA 99164-6430 USA; 4 Key Laboratory of Hangzhou City for Ecosystem Protection and Restoration, College of Life and Environmental Sciences, Hangzhou Normal University, Hangzhou, 310036 China

**Keywords:** Flavonoids, Germplasm resources, Metabolite, Metabolomics, *Taxus*, Taxol

## Abstract

**Background:**

Plants of the genus *Taxus* have attracted much attention owing to the natural product taxol, a successful anti-cancer drug. *T. fuana* and *T. yunnanensis* are two endangered *Taxus* species mainly distributed in the Himalayas. In our study, an untargeted metabolomics approach integrated with a targeted UPLC-MS/MS method was applied to examine the metabolic variations between these two *Taxus* species growing in different environments.

**Results:**

The level of taxol in *T. yunnanensis* is much higher than that in *T. fuana*, indicating a higher economic value of *T. yunnanensis* for taxol production. A series of specific metabolites, including precursors, intermediates, competitors of taxol, were identified. All the identified intermediates are predominantly accumulated in *T. yunnanensis* than *T. fuana*, giving a reasonable explanation for the higher accumulation of taxol in *T. yunnanensis*. Taxusin and its analogues are highly accumulated in *T. fuana*, which may consume limited intermediates and block the metabolic flow towards taxol. The contents of total flavonoids and a majority of tested individual flavonoids are significantly accumulated in *T. fuana* than *T. yunnanensis*, indicating a stronger environmental adaptiveness of *T. fuana*.

**Conclusions:**

Systemic metabolic profiling may provide valuable information for the comprehensive industrial utilization of the germplasm resources of these two endangered *Taxus* species growing in different environments.

**Electronic supplementary material:**

The online version of this article (10.1186/s12870-018-1412-4) contains supplementary material, which is available to authorized users.

## Background

Plants of the genus *Taxus*, coniferous trees or shrubs known as yews, are major sources for various bioactive compounds, such as paclitaxel and its derivatives [1]. The generic paclitaxel, is marketed under the registered trade name Taxol, which is one of the most successful anti-cancer drugs utilized in the past years [2]. The market demand for taxol exceeds the supply, which is limited by several restrictive conditions, such as the slow growth of wild yews, the low taxoid contents and the complex synthesis routes [3].

The biosynthetic pathways of Taxol have been revealed, resulting in a large number of precursors, intermediates and derivatives of Taxol [2]. Firstly, the plastidial 2-C-methyl-D-erythritol phosphate pathway supplies 3 units of the C_5_ isoprenoid precursor isopentenyl diphosphate and one unit of dimethylallyl diphosphate to synthesize the precursor of the diterpenoid taxane core geranylgeranyl diphosphate [4, 5]. The formation of the taxane skeleton is initiated by the cyclization of geranylgeranyl diphosphate yielding taxa-4(5),11(12)-diene, which is mediated by the important enzyme taxadiene synthase [6, 7]. Then, several intermediate enzymatic reactions, such as hydroxylation, acetylation and *N*-benzoylation, occur in the pathway [2]. For example, the acetylation of 10-deacetylbaccatin III (10-DAB) is an essential step to produce baccatin III, which is an advanced precursor for Taxol biosynthesis [8, 9]. Thereafter, the C_13_-side chain is appended to baccatin III to form *N*-debenzoyl-2′-deoxytaxol [10, 11]. The final step of Taxol biosynthesis is conducted by the side chain *N*-benzoyl transferase using *N-*debenzoyltaxol as the actual substrate [12].

Over 500 taxane diterpenoid compounds, including cephalomannine, 7-epi 10-desacetyl paclitaxel and 7-epipaclitaxel, have been identified [13–15]. Recently, novel taxoids have been isolated from different *Taxus* species. For example, taxezopidine J and taxuspine D from the seeds of *T. cuspidata*, a new taxane glucoside from the bark of *T. mairei*, two new rearranged taxane diterpenoids from the barks of *T. wallichiana*, and three new taxane diterpenoids, namely baccatin VIII, IX and X, from the seeds of *T. yunnanensis* were identified over the last two decades [16–19]. Interestingly, intermediate oxygenation steps produce a broad range of taxusin-like metabolites, which are considered as dead-end metabolites and cannot be used for Taxol biosynthesis [20, 21].

An untargeted metabolome provides a shortcut to systematically analyze and compare the features of primary and secondary metabolites among species [22]. Studies of the metabolomes in the *Taxus* genus have been carried out since 2003, profiling the differential metabolites of *T. media* cultures with or without methyl jasmonate treatments [23]. In 2009, the defense mechanism of *T. cuspidata* cells to shear stress was uncovered using a metabolic profiling tool [24]. Fluctuations in the synthesis of taxoids in cultured seedlings of *T. mairei* have also been investigated using a metabolomics approach [25]. The crucial network that controls the biosynthesis of Taxol and its precursors under methyl jasmonate treatments was elucidated by metabolomics profiling data [26]. An integrated metabolomics and transcriptomics analysis of *T. chinensis* cells under different dissolved oxygen conditions suggests a relationship between the changes in taxane production caused by the shift in dissolved oxygen and the global variations in the cell’s central carbon metabolism [27]. Recently, an integrated proteomics/metabolomics analysis revealed an important role of a short-term high doses of ultraviolet (UV)-A radiation in promoting Taxol production in *T. mairei* [28].

To date, ~ 14 species of the *Taxus* genus have been identified; however, the levels of accumulated taxoids can vary significantly among different species and cultivars [29, 30]. *T. fuana* and *T. yunnanensis* are two slow-growing and endangered species mainly distributed in southwestern China [31]. Investigations into the metabolic features contributing to the interspecific differential accumulation of taxoids, as well as other secondary metabolites, between these two morphologically similar *Taxus* species will enhance the highest-yielding species breeding and reveal the mechanism underlying both of the species- and environment- dependent metabolic variations. Furthermore, the Himalayas is one of few paradises of rare vegetation in the world. Our study may provide valuable information for the conservation of these germplasm resources of these two endangered *Taxus* species.

## Methods

### Plant materials

**Fig. 1:**
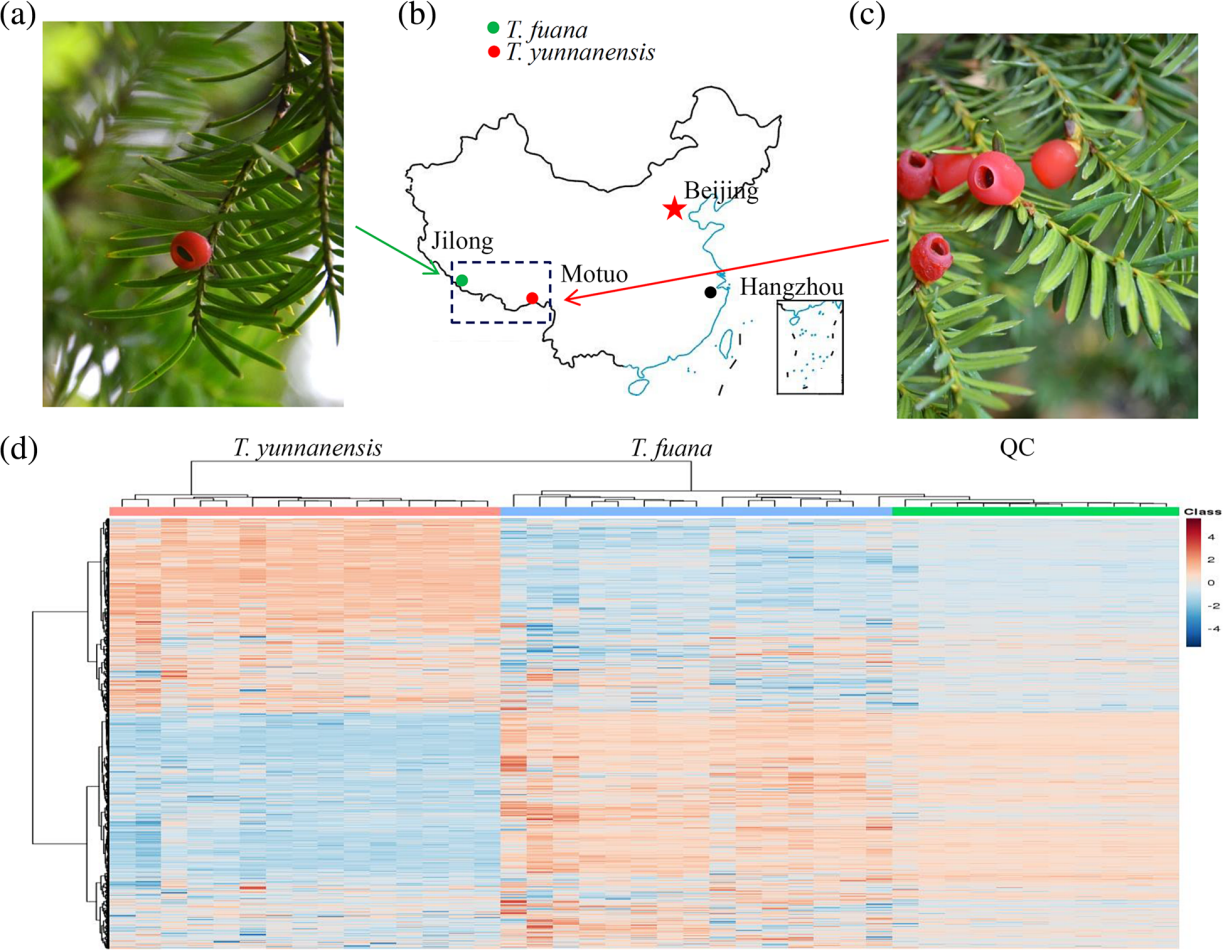
Untargeted metabolite profiling reveals the variations in the abundance of metabolites between *T. yunnanensis* and *T. fuana*. **a** A picture of the twigs of *T. fuana*. **b** The collection sites of *T. fuana* (Jilong) and *T. yunnanensis* (Motuo) were indicated by green and red small dots, respectively. **c** A picture of the twigs of *T. yunnanensis*. **d** A heatmap of the metabolites identified in the metabolomes of the two *Taxus* species (*n* = 15). The heatmap scale ranges from − 4 to + 4 on a *log*_*2*_ scale

Fresh twig samples were harvested from 15 independent 10-year-old wild *Taxus* trees of *T. fuana* in Jilong (29° 22’ N, 95° 26′ E, Tibet, China) and *T. yunnanensis* in Motuo (28° 28’ N, 85° 13′ E, Tibet, China), respectively (Fig. 1a). The average altitude of Jilong is 4,000 m and the average altitude of Motuo is 1,200 m.

### Standards and reagents

Paclitaxel (PTX; ≥99%), 10-deacetylbaccatin III (10-DAB; ≥98%), baccatin III (BAC; ≥99%), apigenin (API; ≥98%), quercetin (QUE; ≥98.5%), luteolin (LUT; ≥98%), kaempferol (KAE; ≥98%), (+)-catechin (CAT; ≥97%) and (−)-epicatechin (E-CAT; ≥98%) were obtained from Aladdin Biochemical Technology (Shanghai, China). 10-Deacetyl paclitaxel (DAP; 98%), 7-epipaclitaxel (7-E-PTX; 98%), amentoflavone (AMF; 98%) and ginkgetin (GKG; 98%) were purchased from J&K Scientific (Beijing, China). 7-Epi 10-desacetyl paclitaxel (7-E-DAP) was obtained from Toronto Research Chemicals (Toronto, Canada). Cephalomannine (CEP; ≥98.5%) was provided by MUST Bio-Technology (Chengdu, China). Methanol (HPLC grade) and formic acid (HPLC grade) were obtained from Merck (Kenilworth, NJ, USA).

### Metabolite extraction for the untargeted metabolomic analysis

The twig samples from the two *Taxus* species (25 mg each, *n* = 15) were transferred to a microcentrifige tube. An aliquot of 800 μL pre-cooled 50% methanol was added to the tube. The mixture was ground at a rate of 1,900 strokes/min for 2 min using a 2010 Geno/Grinder (SPEX SamplePrep, Metuchen, NJ, USA). The samples were mixed with 500 μL of pre-cooled chloroform/methanol/water (v:v:v, 1:3:1), vortexed at 4 °C in the dark for 15 min, and subjected to ultrasonication for 5 min on ice. After centrifugation at 13,000 g for 15 min at 4 °C, the supernatant was transferred to a new tube. The extraction solution was vacuum-dried and resuspended in 50% methanol. Meanwhile, a quality control (QC) sample (a pool of all the samples) was prepared by mixing an equal volume of each extraction sample from one *Taxus* species. All the samples were stored at − 80 °C prior to Ultra-performance liquid chromatography (UPLC)-MS/MS analysis.

### UPLC-MS/MS analysis for the untargeted metabolomics

The chromatographic separations of all the samples were performed using a SCIEX UPLC system (Applied Biosystems, Foster City, CA, USA). An ACQUITY BEH Amide column (2.1 × 100 mm, 1.7 μm particle size; Waters, Milford, MA, USA) was used for the reversed-phase separation. The column oven was maintained at 35 °C and the flow rate was 0.4 mL/min. The mobile phase consisted of solvent A (aqueous solution with 25 mM ammonium acetate and 25 mM NH_4_H_2_O) and solvent B (IPA:ACN = 9:1 + 0.1% formic acid). The gradient elution procedures were set as follows: 0 ~ 0.5 min, 95% solvent A; 0.5 ~ 9.5 min, 95% to 65% solvent A; 9.5 ~ 10.5 min, 65% ~ 40% solvent A; 10.5 ~ 12.0 min, 40% solvent A; 12.0 ~ 12.2 min,40% ~ 95% solvent A; 12.2 ~ 15.0 min, 95% solvent A. The injection volume for each sample was 4 μL.

A high-resolution tandem mass spectrometer SCIEX Triple-TOF-5600 plus (Applied Biosystems) was used to detect metabolites eluted from the column. The Q-TOF was operated in both positive and negative ion modes. The curtain gas was set at a pressure of 30 psi, ion source gas 1 and ion source gas 2 were both set at 60 psi, and the interface heater temperature was set at 650 °C. The ion spray voltages were set at 5000 V and - 4500 V for the positive and negative ion modes, respectively. The mass spectrometry data were acquired in information-dependent acquisition (IDA) mode. The TOF mass range was from 60 to 1,200 Da. The survey scans were acquired over 150 ms and as many as 12 product ion scans were collected if exceeding a threshold of 100 counts per second and with a 1+ charge-state. The total cycle time was held constant at 0.56 s. Four time bins were summed for each scan at a pulser frequency value of 11 kHz by monitoring of the 40 GHz multichannel time-to-digital converter (TDC) detector with four-anode/channel detection. The dynamic exclusion was set to 4 s. During the acquisition, the mass accuracy was calibrated every 20 samples. Furthermore, in order to evaluate the system stability of UPLC-MS/MS analysis, a QC sample was analyzed right after every 10 samples.

### Bioinformatic analysis of the untargeted metabolomic dataset

The acquired MS data pretreatments including peak picking, peak grouping, retention time correction, second peak grouping, and annotation of isotopes and adducts were performed using the XCMS software. LC−MS raw data files were converted into mz.XML format and then processed by the XCMS, CAMERA and metaX toolbox implemented with the R software. Each ion was identified by the retention time (RT) and *m/z* data. Intensities of each peak were recorded and a three dimensional matrix containing arbitrarily assigned peak indices (retention time-*m/z* pairs), sample names (observations) and ion intensity information (variables) was generated.

The online KEGG, HMDB databases were used to annotate the metabolites by matching the exact molecular mass data (*m/z*) of the samples with those from the databases. If a mass difference between the observed value and the database value was less than 10 ppm, the metabolite would be annotated and the molecular formula of the metabolite would further be identified and validated by the isotopic distribution measurement. In addition, an in-house fragment spectrum library of metabolites was also used to validate the metabolite identification.

The intensities of peak data were further preprocessed by an in-house software metaX. Those features that were detected in less than 50% of the QC samples or 80% of the biological samples were removed, and the remaining peaks with missing values were imputed with the k-nearest neighbor algorithm to further improve the data quality. Principal component analysis (PCA) was performed for outlier detection and batch effect evaluation using the pre-processed dataset. Quality control-based robust LOESS signal correction was fitted to the QC data with respect to the order of injection to minimize signal intensity drift over time. In addition, the relative standard deviations of the metabolic features were calculated across all the QC samples, and those > 30% were then removed.

### Quantitative analysis of taxoids and flavonoids

Twigs were harvested from six trees of *T. fuana* and *T. yunnanensis*, respectively. The samples were thoroughly dried at 40 °C and then ground into powder. A modified version of a previously published method was used to prepare crude extracts [32]. In brief, the powder was passed through a filter (mesh size 0.42 mm) and 0.5 g of the fine powder was precisely weighed, and mixed with 15 mL of 100% methanol. The mixture was ultrasonicated at 150 W and 40 kHz for 30 min. After centrifugation at 5000 rpm for 5 min, the supernatant was collected. The residue was re-suspended in 15 mL methanol and subjected to the same extraction procedures. After centrifugation, the second supernatant was combined with the first extract. Before UPLC-MS/MS analysis, the crude *Taxus* extracts were diluted at a ratio of 1:10 and all the samples were passed through 0.22 μm membrane filters.

The quantification of seven taxoids and eight flavonoids was carried out using an LC-30 AD UPLC system (Shimadzu, Japan) coupled with a SCIEX QTRAP 6500 mass spectrometer (Applied Biosystems). The MultiQuant software (version 3.0) was used for data acquisition and processing.

To separate seven taxoids, the UPLC was performed on a Kinetex C18 column (100 × 4.6 mm, 2.6 μm; Phenomenex, Torrance, CA, USA). The mobile phase consisted of 65% solvent A (methanol) and 35% solvent B (aqueous solution with 0.1% formic acid and 2 mM ammonium formate). The flow rate was set at 0.2 mL/min. The column oven was maintained at 30 °C and the injection volume was 5 μL. The capillary temperature was set at 270 °C and the ion spray voltage was set at 3.0 kV. Multiple reaction monitoring (MRM) was applied for the quantification in the positive ionization mode. The transitions of *m/z* 876.4 → 308.2, 567.4 → 445.3, 609.5 → 427.3, 834.4 → 308.2, 832.3 → 264.1, 876.4 → 591.4 and 834.4 → 308.2 were used for the quantification of PTX, 10-DAB, BAC, DAP, CEP, 7-E-PTX and 7-E-DAP, respectively.

The separation of eight flavonoids was achieved on an ACQUITY UPLC BEH C18 column (2.1 × 100 mm, 1.7 μm; Waters). The gradient elution was performed with solvent A (methanol) and solvent B (aqueous solution with 0.1% formic acid and 2 mM ammonium formate) at a flow rate of 0.3 mL/min using the following programs: 0 ~ 2.0 min, 5% solvent A; 2.0 ~ 3.0 min, 5 ~ 35% solvent A; 3.0 ~ 10.0 min, 35 ~ 50% solvent A; 10.0 ~ 13.0 min, 50 ~ 65% solvent A; 13.0 ~ 14.5 min, 65% solvent A; 14.5 ~ 15.0 min, 65 ~ 90% solvent A; 15.0 ~ 17.0 min, 90% solvent A; 17.0 ~ 17.1 min, 90 ~ 5% solvent A; 17.1 ~ 20.0 min, 5% solvent A. The column oven was maintained at 40 °C and the injection volume was 5 μL. The determination of QUE, LUT, KAE, AMF and GKG was performed in the negative ion mode. The transitions of *m/z* 301.1 → 151.0, 285.0 → 133.0, 285.1 → 159.0, 537.0 → 374.9 and 565.2 → 533.1 were used for the quantification of QUE, LUT, KAE, AMF and GKG, respectively. The measurement of CAT, E-CAT and API was performed in the positive ion mode. The transitions of *m/z* 291.1 → 139.0, 291.0 → 139.0 and 271.0 → 153.0 were used for the quantification of CAT, E-CAT and API, respectively.

### Statistical analysis

For the untargeted metabolomic analysis, Wilcoxon tests were carried out to examine metabolic differences between every two samples. The *P* values were adjusted for multiple testing correction by false discovery rate (FDR; Benjamini–Hochberg). The supervised partial least squares-discriminant analysis (PLS-DA) was conducted using the metaX to discriminate different variables between groups. The VIP value was calculated and a VIP cut-off value of 1.0 was used to select important features.

The quantification results of taxoids and flavonoids are presented as mean ± SD (*n* = 6). The statistical analysis was performed using SPSS software (version 19.0; SPSS Inc., Chicago, IL, USA). A one-way analysis of variance (ANOVA) was carried out to compare the content differences of taxoids and flavonoids between *T. fuana* and *T. yunnanensis*. A *P* value < 0.05 was considered statistically significant.

## Results

### Untargeted metabolite profiling of two endangered *Taxus* species

To explore global metabolic variations, an untargeted metabolomic approach was used (*n* = 15), which identified 4,986 annotated metabolites from 7,251 ion features (Additional file 1). To examine the quality of the acquired MS data, total ion chromatograms (TICs) were generated for all of the samples and revealed a high degree of overlap (Additional file 2). In addition, the *m/z* width and retention-time width analyses suggested that the sample preparation and instrument state met the required standards (Additional file 3). An overview of the metabolite profiling of *T. fuana* and *T. yunnanensis* is shown in Fig. 1d. Unlike the similarity in twig morphology, dramatic variations in the metabolomes between *T. fuana* and *T. yunnanensis* were observed.

Based on their annotations, a number of metabolites were assigned to at least one primary or secondary metabolic category. The top 20 largest metabolic categories, such as porphyrin and chlorophyll metabolism (134 metabolites), 2-oxocarboxylic acid metabolism (134 metabolites), biosynthesis of amino acids (127 metabolites), ABC transporters (126 metabolites), diterpenoid biosynthesis (103 metabolites), and other secondary metabolites (141 metabolites) are shown in Additional file 4 and Additional file 5.

### Metabolite profiling reveals variations in the abundance levels of metabolites between *T. fuana* and *T. yunnanensis*

To provide a deep overview of the metabolic variations, several quality control parameters for the quantification, including coefficient of variation (CV), principal component (PC) and normalized intensity, were analyzed. The CV values were lower than 30%, suggesting a good repeatability (Additional file 6). The PC analysis showed that PC1 and PC2 were responsible for 59.75% and 5.43% of the variation, respectively, indicating a clear separation between these two *Taxus* species (Additional file 7). After filtering, 4,986 high-quality metabolites were used to screen the differentially accumulated metabolites (DAMs). The statistical analysis identified 1,972 significant DAMs, including 788 *T. yunnanensis* predominantly accumulated metabolites and 1,184 *T. fuana* predominantly accumulated metabolites (Additional file 8).

**Fig. 2:**
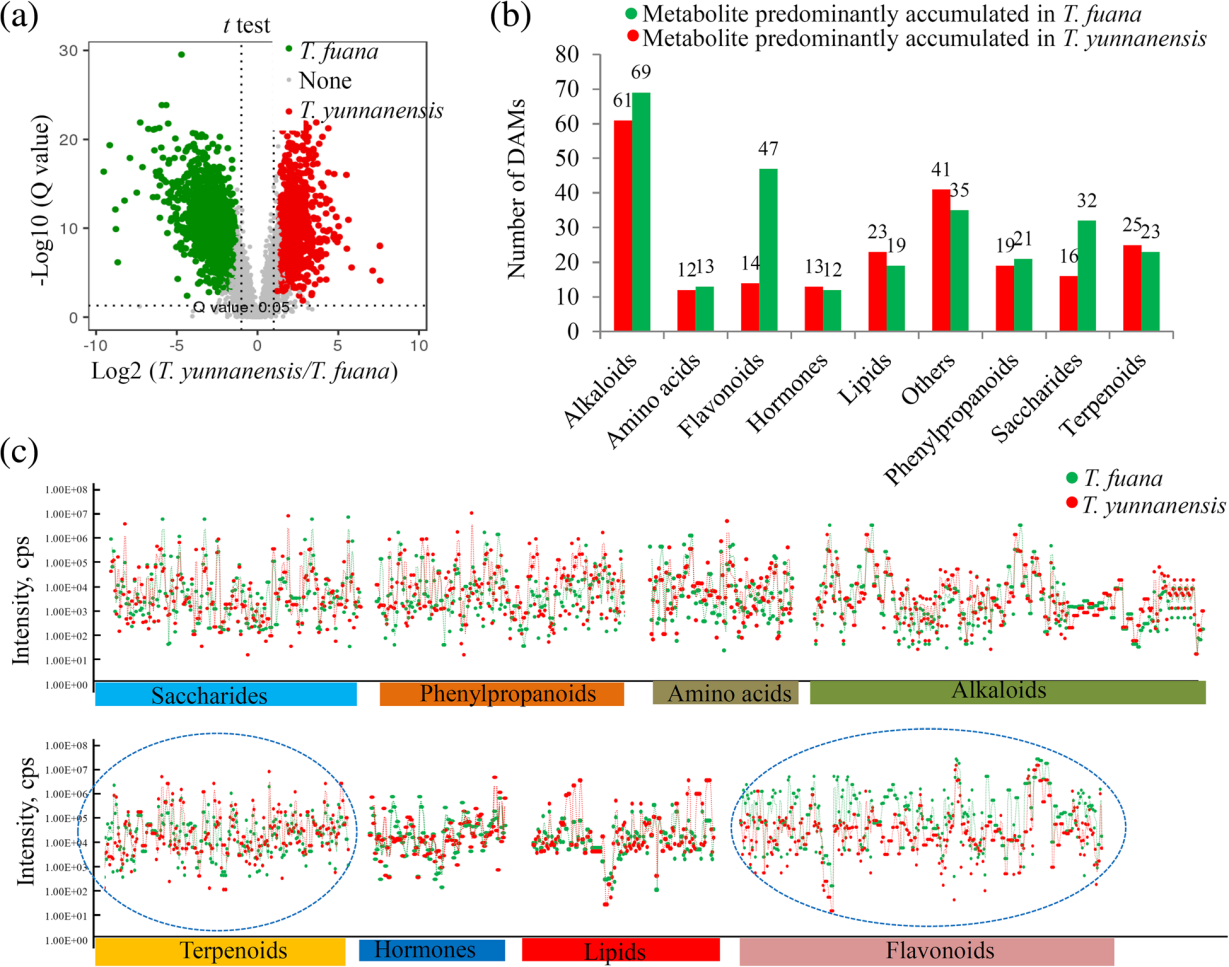
Identification of the DAMs between *T. fuana* and *T. yunnanensis*. **a** Significance analysis of the DAMs between the two *Taxus* species by Volcanoplot. **b** The numbers of *T. yunnanensis* predominantly accumulated metabolites and *T. fuana* predominantly accumulated metabolites in each metabolic category. **c** The abundances of the DAMs belonging to different metabolic categories

All of the DAMs were assigned to various major metabolic categories, including alkaloids, amino acids, flavonoids, hormones, lipids, terpenoids, phenylpropanoids, saccharides, and others. For most of these categories, the numbers of metabolites predominantly accumulated in *T. fuana* were similar to those in *T. yunnanensis*. For instance, the number of *T. fuana*-predominantly accumulated terpenoids was similar to that of *T. yunnanensis*-predominantly accumulated terpenoids (Fig. 2b). However, 47 flavonoids were predominantly accumulated in *T. fuana*, while only 14 flavonoids were predominantly accumulated in *T. yunnanensis*. The normalized ion intensity of each metabolite belonging to these categories was calculated and is shown in Fig. 2c.

### Untargeted metabolomics analysis reveals variations in the precursors, intermediates and competitors of Taxol

**Fig. 3:**
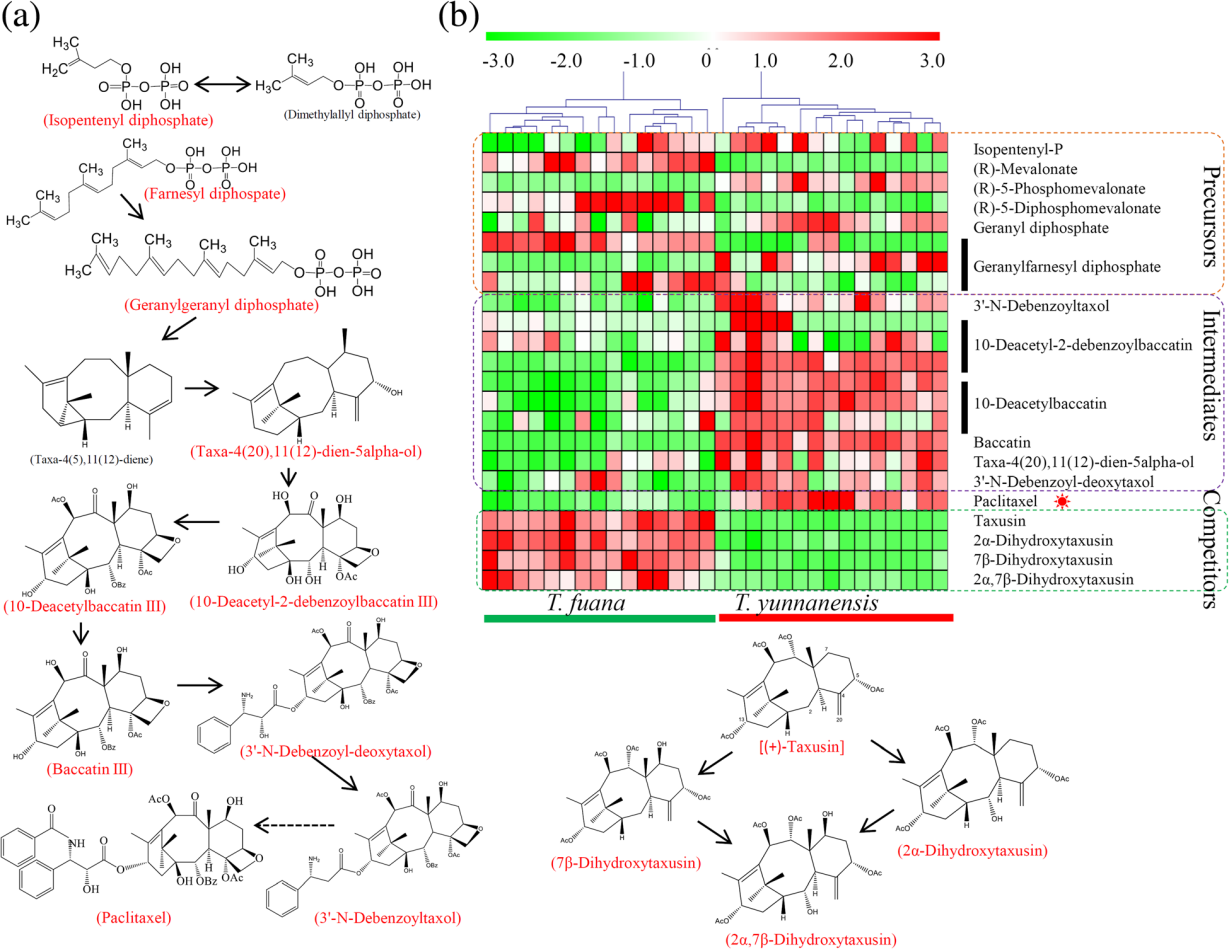
Variations in the abundances of the precursors, intermediates, competitors of taxol between *T. fuana* and *T. yunnanensis*. **a** An overview of the taxol biosynthesis pathway. **b** A heatmap of the relative amounts of the precursors, intermediates, competitors of taxol in the two *Taxus* species. The precursors from MEP pathway were included in a red box, the intermediates and derivatives were included in a purple box, and the competitors of taxol were included in a green box. The heatmap scale ranges from − 3 to + 3 on a *log*_*2*_ scale

Taxol biosynthesis is a complicated metabolic pathway that involves a large number of secondary metabolites, including the precursors, intermediates and competitors of Taxol (Fig. 3a) [2]. After searching the metabolite pool, six important precursors from the 2-C-methyl-D-erythritol phosphate pathway, isopentenyl diphosphate, geranyl diphosphate and geranylfarnesyl diphosphate, six intermediates, 3’-*N*-debenzoyltaxol, 10-deacetyl-2-debenzoylbaccatin III, 10-deacetylbaccatin III, taxa-4(5),11(12)-dien-5α-ol, 3’-*N*-debenzoyl-deoxytaxol, and baccatin III, paclitaxel, and four Taxol competitors, taxusin, 2α-dihydroxytaxusin, 7β-dihydroxytaxusin, and 2α,7β-dihydroxytaxusin, were identified. There were no significant differences in the accumulations of the precursors between *T. fuana* and *T. yunnanensis*; however, paclitaxel was predominantly accumulated in *T. yunnanensis*. Intereastingly, most of the intermediates were highly accumulated in *T. yunnanensis* while all of the taxusin-like metabolites were significantly accumulated in *T. fuana* (Fig. 3b).

### Quantitative analysis confirms the variations in taxoids

**Fig. 4:**
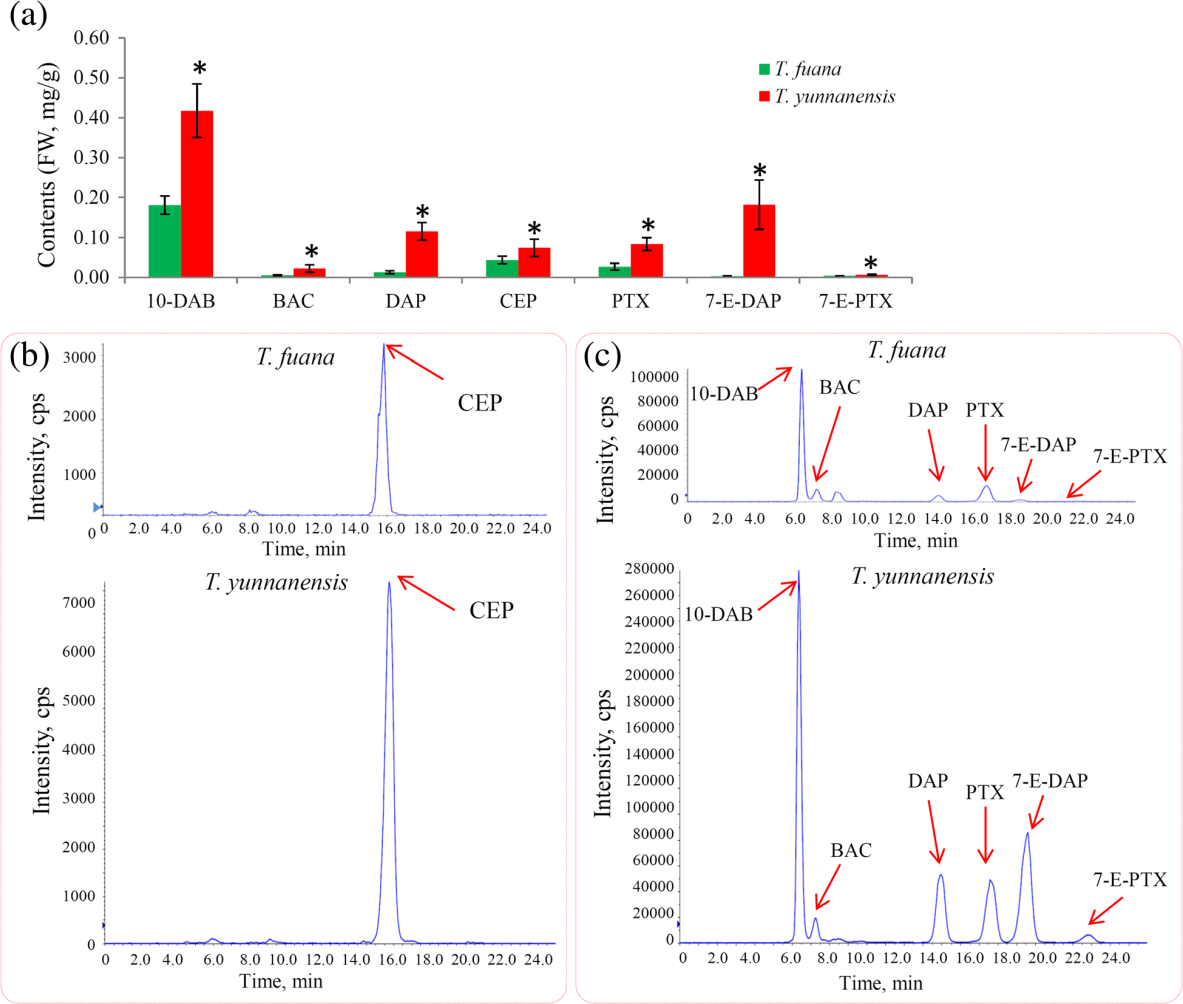
Determination of the contents of taxoids in *T. fuana* and *T. yunnanensis*. **a** The contents of paclitaxel and six intermediates were quantified by a UPLC-MS/MS method. A *P* value < 0.001 was considered to be statistically significant and indicated by “*”. **b** Representative chromatograms of cephalomannine in *T. yunnanensis* and *T. fuana* in the positive ion mode. **c** Representative TIC chromatograms of six taxoids in *T. yunnanensis* and *T. fuana* in the positive ion mode. 10-DAB:10-deacetylbaccatin III; BAC: baccatin III; DAP: 10-deacetylpaclitaxel; CEP: cephalomannine; PTX: paclitaxel; 7-E-DAP: 7-epi 10-desacetyl paclitaxel; and 7-E-PTX: 7-epipaclitaxel

To precisely determine the content differences of Taxol and its derivatives between *T. fuana* and *T. yunnanensis*, seven taxoids, including Taxol, cephalomannine, baccatin III, 10-deacetylbaccatin III (10-DAB), 10-deacetylpaclitaxel (DAP), 7-epi 10-desacetyl paclitaxel (7-E-DAP) and 7-epipaciltaxel (7-E-PTX), were quantified using a UPLC-MS/MS method. The untargeted metabolomics analysis indicated that paclitaxel was predominantly accumulated in *T. yunnanensis*, and the direct quantification with an authentic standard of paclitaxel confirmed this phenomenon. The content of taxol in *T. yunnanensis* (0.084 mg/g) was 3.1-fold greater than that in *T. fuana* (0.027 mg/g). Furthermore, all the six other taxoids were significantly accumulated in *T. yunnanensis* compared with in *T. fuana* (Fig. 4a). The representative TIC chromatograms of these taxoids between *T. fuana* and *T. yunnanensis* are shown in Fig. 4b and c.

### Untargeted metabolomics analysis reveals variations in the flavonoids

**Fig. 5:**
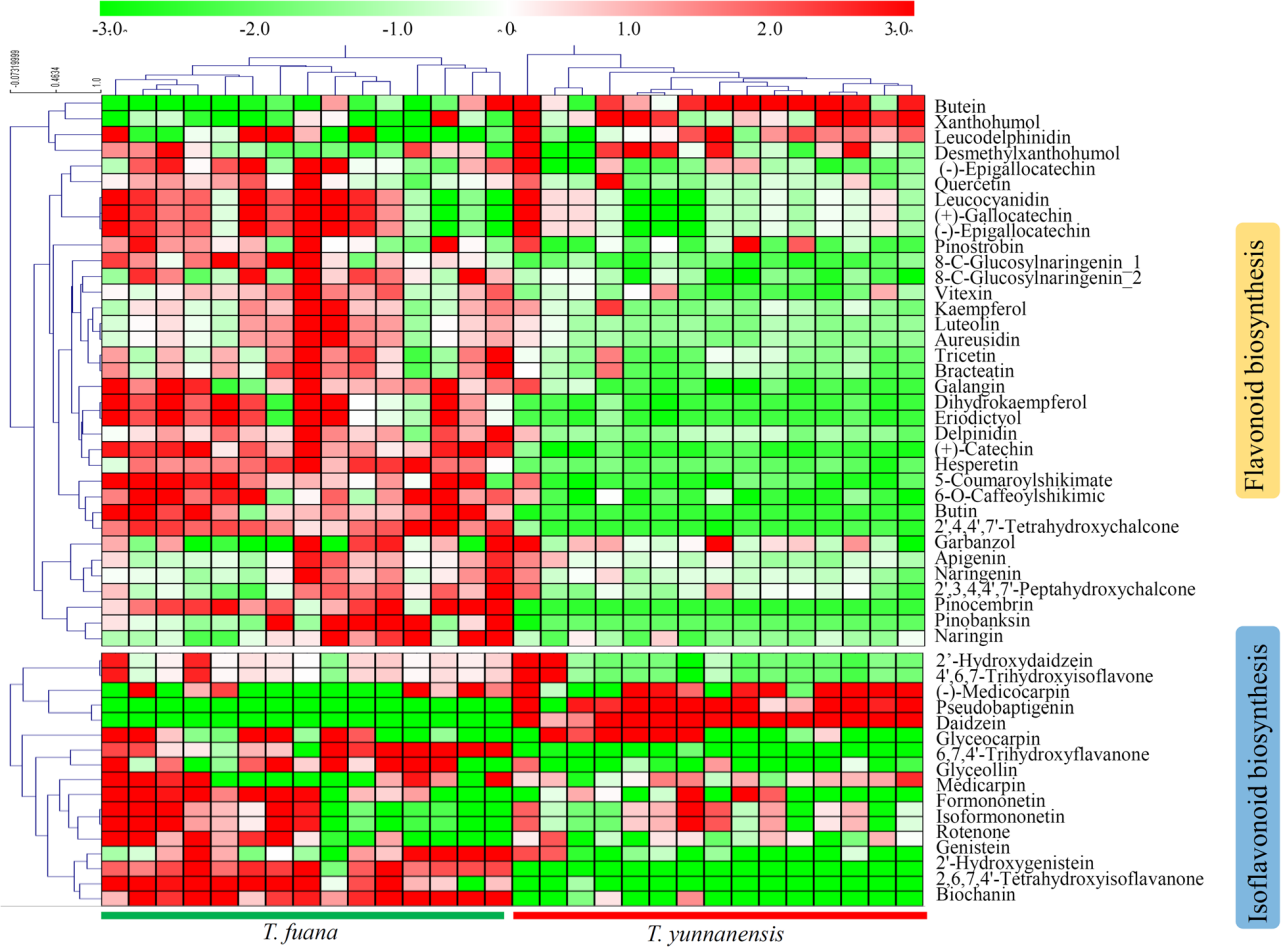
Untargeted metabolomic analysis reveals the variations in the flavonoids between *T. fuana* and *T. yunnanensis*. A heatmap of the relative amounts of flavonoids in the two *Taxus* species. The heatmap scale ranges from − 3 to + 3 on a *log*_*2*_ scale

A large number of flavonoids have been isolated from various *Taxus* species to date. In our study, 35 metabolites involved in flavonoid biosynthesis and 17 metabolites involved in isoflavonoid biosynthesis were identified. For flavonoid biosynthesis, most of the metabolites, except for butein, xanthohumol, and leucodelphinidin, were significantly accumulated in *T. fuana* compared with in *T. yunnanensis*. For isoflavonoid biosynthesis, most of the metabolites, except for (−)-medicocarpin, pseudobaptigenin, and daidzein, were predominantly accumulated in *T. fuana* compared with in *T. yunnanensis* (Fig. 5).

### Quantitative analysis confirms variations in flavonoids

**Fig. 6:**
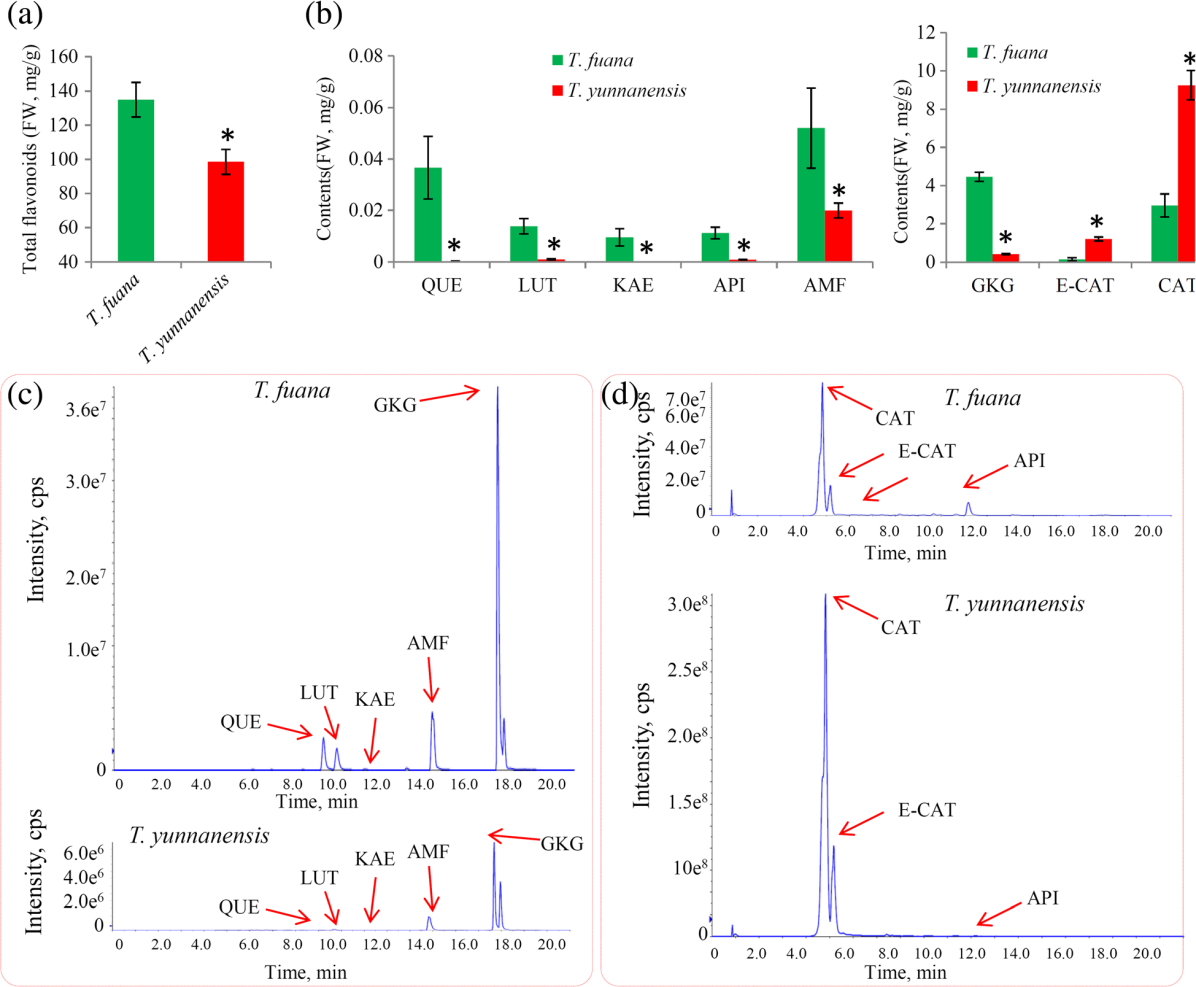
Determination of the contents of total and individual flavonoids in *T. fuana* and *T. yunnanensis*. **a** The contents of total flavonoids in *T. fuana* and *T. yunnanensis*. **b** The contents of eight individual flavonoids in *T. fuana* and *T. yunnanensis*. A *P* value < 0.001 was considered to be statistically significant and indicated by “*”. **c** Chromatograms of individual flavonoids in negative mode. **d** Chromatograms of individual flavonoids in positive mode. CAT: (+)-Catechin; E-CAT: (−)-Epicatechin; API: Apigenin; QUE: Quercetin; LUT: Luteolin; KAE: Kaempferol; AMF: Amentoflavone; and GKG: Ginkgetin

The levels of total flavonoids and eight individual flavonoids, including apigenin, luteolin, kaempferol, quercetin, (+)-catechin, (−)-epicatechin, amentoflavone and ginkgetin in *T. fuana* and *T. yunnanensis* were determined. The total flavonoid contents were significantly higher in *T. fuana* than in *T. yunnanensis* (Fig. 6a). Similarly, most of the individual flavonoids, including apigenin, luteolin, kaempferol, quercetin, amentoflavone and ginkgetin, were predominantly accumulated in *T. fuana* than *T. yunnanensis*. Only two flavonoids, (+)-catechin and (−)-epicatechin, were predominantly accumulated in *T. yunnanensis* rather than in *T. fuana* (Fig. 6b). The representative TIC chromatograms of these flavonoids in *T. fuana* and *T. yunnanensis* samples are shown in Fig. 6c and d.

## Discussion

Trees from the *Taxus* genus are major natural resources for Taxol extraction [28]. To date, a large number of secondary metabolites, including taxane diterpenoids and flavonoids, have been isolated and identified in various *Taxus* species [13, 33]. *T. fuana* and *T. yunnanensis* are two endangered *Taxus* species sparsely distributed in southern Tibet and western Yunnan Province, China [34, 35]. In past decades, deforestation has driven these *Taxus* trees to the edge of extinction [36]. A metabolomics approach provides a good opportunity to systematically analyze the metabolic variations between *T. fuana* and *T. yunnanensis*, which grow in different environments. Our data will enhance the conservation of the germplasm resources of these endangered *Taxus* species [28].

A traditional metabolomics analysis could only identify a small number of metabolites. For example, only 65 intracellular metabolites were identified and quantified in *T. cuspidata* cells under laminar shear stress [24]. A metabolomics approach using LC-IT-TOF-MS identified 10 compounds in the cultured seedlings of *T. mairei* [25]. In 2014, a metabolomics dataset identified 12 metabolites in methyl jasmonate-induced *T. chinensis* cells [26]. In our study, a large number of metabolites, belonging to various metabolic pathways, were identified (4986 annotated metabolites), which is more than the previously published metabolomes. Embracing comprehensive metabolite profiling allowed us to explore new metabolites and potential interactions, directly or indirectly, among the metabolites in the *Taxus* genus.

The accumulation of metabolites is a complex and variable trait largely affected by genetic and environmental factors [37, 38]. *T. fuana* and *T. yunnanensis* are distributed in different regions of the Yalu Tsangpo river basin, and dramatic variations in the metabolomes between these *Taxus* species were observed (Fig. 1d) [39]. Our data showed that the Taxol level in *T. yunnanensis* was more than three times greater than that in *T. fuana*, indicating the greater economic value of *T. yunnanensis* for paclitaxel production. A series of specific metabolites, including precursors, intermediates, and competitors of Taxol, were identified in our study. No significant differences in the precursors between *T. fuana* and *T. yunnanensis* were observed, suggesting that the precursor supply is not a limiting factor in the differential accumulation of Taxol [2]. In the biosynthesis pathway of taxol, the oxygenation, hydroxylation, benzoylation and acetylation steps produce many intermediates [7, 40]. In our study, six important intermediates were identified (Fig. 3). P450-mediated hydroxylation of taxa-4(5),11(12)-diene to taxa-4(5),11(12)-dien-5α-ol is the first oxygenation step after the formation of the taxane skeleton [40, 41]. 10-DAB is a natural compound isolated from the needles of the *Taxus* species, and the conversion of taxane glycosides to 10-DAB improves the total yield of paclitaxel [42, 43]. The final acylation step is the conversion of 3’-*N*-debenzoyltaxol to mature Taxol by 3’-*N*-debenzoyltaxol *N*-benzoyltransferase [44]. Interestingly, all of these intermediates were predominantly accumulated in *T. yunnanensis* compared with in *T. fuana*, suggesting a reasonable explanation for the higher accumulation of Taxol in *T. yunnanensis*.

Taxusin and its analogues, the prominent metabolites of yew heartwood, are considered to be dead-end metabolites for Taxol biosynthesis [45]. Taxusin-like metabolites, such as 2α-hydroxytaxusin, 7β-hydroxytaxusin and 2α,7β-dihydroxytaxusin, have been used as surrogates in studies of the intermediate oxygenation steps of the biosynthesis pathway [20]. In our study, the accumulations of taxusin and its analogues were significantly higher in *T. fuana* than in *T. yunnanensis*. The high accumulation levels of taxusin and taxusin-like metabolites may consume limited intermediates and block the metabolic flow toward Taxol.

The taxoid and flavonoid contents were greatly affected by environmental factors, such as temperature, altitude and light [13, 28, 46]. In our study, the comprehensive analysis of the metabolites highlighted significant variations in the contents of various flavonoids (Fig. 5a). In general, flavonoids function as antioxidants in plants and can be induced by abiotic and biotic stresses [47, 48]. For example, the contents of flavonoids were elevated in *Taxus* trees under UV-B radiation [49]. Jilong (with an average altitude of 4,000 m) has a more extreme environment than Motuo (with an average altitude of 1,200 m) (www.chenwentan.com/). Consequently, the higher accumulation of flavonoids suggested a stronger environmental adaptiveness of *T. fuana* (collected from Jilong) than *T. yunnanensis* (collected from Motuo) [50]. Eastern Himalayan populations of *T. wallichiana*, located at higher altitudes, tend to accumulate more paclitaxel than populations from lower altitudes [51]. However, *T. fuana* from higher altitudes accumulated less paclitaxel than *T. yunnanensis* from lower altitudes, suggesting that paclitaxel accumulation is controlled by both hereditary and environmental factors.

## Conclusions

In our study, a metabolomics approach was applied to examine the metabolic variations in the twigs between *T. fuana* and *T. yunnanensis*, which are two endanger species growing in different environments. In total, 4,986 annotated metabolites belonging to various metabolic categories were identified. The level of taxol in *T. yunnanensis* is significantly higher than that in *T. fuana*. All the identified intermediates for taxol biosynthesis are predominantly accumulated in *T. yunnanensis* while four competitors for taxol biosynthesis are predominantly accumulated in *T. fuana*. Furthermore, the contents of total and some individual flavonoids are significantly accumulated in *T. fuana* than *T. yunnanensis*. Our study may provide valuable information for the comprehensive utilization and conservation of the germplasm resources of these two endangered *Taxus* species growing in different environments.

## Additional files

Additional file 1: **Table S1.** Detail information of all identified metabolites. (XLSX 2024 kb)

Additional file 2: **Figure S1.** The total ion chromatograms of all the samples. (DOCX 531 kb)

Additional file 3: **Figure S2.** The m/z width (a) and retention time width (b) of our MS data. (DOCX 611 kb)

Additional file 4: **Figure S3.** The top 20 largest metabolic categories of all identified metabolites. (DOCX 351 kb)

Additional file 5: **Table S2.** Identification and classification of all metabolites. (XLSX 13 kb)

Additional file 6: **Figure S4.** The coefficient of variation of the metabolites from *T. yunnanensis* and *T. fuana*. (DOCX 91 kb)

Additional file 7: **Figure S5.** The principal component analysis of the data from these two *Taxus* species. (DOCX 64 kb)

Additional file 8: **Figure S6.** The statistical analysis of the DAMs between *T. fuana* and *T. yunnanensis*. (DOCX 15 kb)

## Abbreviations

10-DAB: 10-deacetylbaccatin III
ANOVA: Analysis of variance
CV: Coefficient of variation
DAM: Differentially accumulated metabolite
FDR: False discovery rate
IDA: Information-dependent acquisition
MRM: Multiple reaction monitoring
PC: Principal component
PCA: Principal component analysis
QC: Quality control
RT: Retention time
TDC: Time-to-digital converter
UPLC-MS/MS: Ultra Performance Liquid Chromatography-Coupled Mass Spectrometry
