# Supplementary material for: Comparative metabolomics reveals the metabolic variations between two endangered Taxus species (T. fuana and T. yunnanensis) in the Himalayas
Source: BMC Plant Biol. 2018 Sep 17;18:197. doi: 10.1186/s12870-018-1412-4 (PMC6142684; doi:10.1186/s12870-018-1412-4)
Supplement: Supplementary file 2 — Figure S1. The total ion chromatograms of all the samples. (DOCX 531 kb) [file 12870_2018_1412_MOESM2_ESM.docx]

Figure S1 The total ion chromatograms of all the samples.
