# Supplementary material for: Comparative metabolomics reveals the metabolic variations between two endangered Taxus species (T. fuana and T. yunnanensis) in the Himalayas
Source: BMC Plant Biol. 2018 Sep 17;18:197. doi: 10.1186/s12870-018-1412-4 (PMC6142684; doi:10.1186/s12870-018-1412-4)
Supplement: Supplementary file 4 — Figure S3. The top 20 largest metabolic categories of all identified metabolites. (DOCX 351 kb) [file 12870_2018_1412_MOESM4_ESM.docx]

Figure S3 The top 20 largest metabolic categories of all identified metabolites.
