# Supplementary figures and images for: Comparative metabolomics reveals the metabolic variations between two endangered Taxus species (T. fuana and T. yunnanensis) in the Himalayas
Source: BMC Plant Biol. 2018 Sep 17;18:197. doi: 10.1186/s12870-018-1412-4 (PMC6142684; doi:10.1186/s12870-018-1412-4)

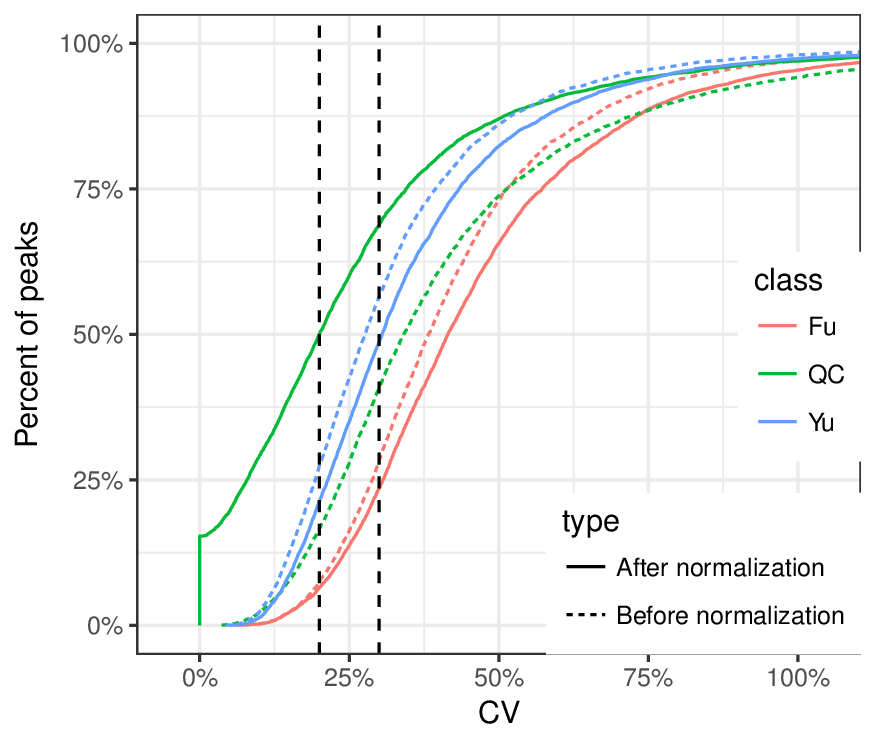


Figure S4 The coefficient of variation of the metabolites from *T. yunnanensis* and *T. fuana*.

Supplement: Supplementary file 6 — Figure S4. The coefficient of variation of the metabolites from T. yunnanensis and T. fuana. (DOCX 91 kb) [file 12870_2018_1412_MOESM6_ESM.docx]
