# Supplementary material for: Comparative metabolomics reveals the metabolic variations between two endangered Taxus species (T. fuana and T. yunnanensis) in the Himalayas
Source: BMC Plant Biol. 2018 Sep 17;18:197. doi: 10.1186/s12870-018-1412-4 (PMC6142684; doi:10.1186/s12870-018-1412-4)
Supplement: Supplementary file 7 — Figure S5. The principal component analysis of the data from these two Taxus species. (DOCX 64 kb) [file 12870_2018_1412_MOESM7_ESM.docx]

Figure S5 The principal component analysis of the data from these two *Taxus* species.
