# Supplementary material for: Comparative metabolomics reveals the metabolic variations between two endangered Taxus species (T. fuana and T. yunnanensis) in the Himalayas
Source: BMC Plant Biol. 2018 Sep 17;18:197. doi: 10.1186/s12870-018-1412-4 (PMC6142684; doi:10.1186/s12870-018-1412-4)
Supplement: Supplementary file 8 — Figure S6. The statistical analysis of the DAMs between T. fuana and T. yunnanensis. (DOCX 15 kb) [file 12870_2018_1412_MOESM8_ESM.docx]

Figure S6 The statistical analysis of the DAMs between *T. fuana* and *T. yunnanensis.*
